# Intelligent Diagnosis towards Hydraulic Axial Piston Pump Using a Novel Integrated CNN Model

**DOI:** 10.3390/s20247152

**Published:** 2020-12-14

**Authors:** Shengnan Tang, Yong Zhu, Shouqi Yuan, Guangpeng Li

**Affiliations:** 1 National Research Center of Pumps, Jiangsu University, Zhenjiang 212013, China; 2111811013@stmail.ujs.edu.cn (S.T.); zhuyong@ujs.edu.cn (Y.Z.); 2221911015@stmail.ujs.edu.cn (G.L.); 2 State Key Laboratory of Fluid Power and Mechatronic Systems, Zhejiang University, Hangzhou 310027, China; 3 Ningbo Academy of Product and Food Quality Inspection, Ningbo 315048, China

**Keywords:** intelligent fault diagnosis, deep learning, convolutional neural network, continuous wavelet transform, hydraulic axial piston pump

## Abstract

As a critical part of a hydraulic transmission system, a hydraulic axial piston pump plays an indispensable role in many significant industrial fields. Owing to the practical undesirable working environment and hidden faults, it is challenging to precisely and effectively detect and diagnose the varying fault in the engineering. Deep learning-based technology presents special strengths in processing mechanical big data. It can simultaneously complete the feature extraction and classification, and achieve the automatic information learning. The popular convolutional neural network (CNN) is exploited for its potent ability of image processing. In this paper, a novel combined intelligent method is developed for fault diagnosis towards a hydraulic axial piston pump. First, the conversion of signals to images is conducted via continuous wavelet transform; the effective feature is preliminarily extracted from the transformed time-frequency images. Second, a novel deep CNN model is constructed to achieve the fault classification. To disclose the potential learning in the disparate layers of the CNN model, the visualization of reduced features is performed by employing *t*-distributed stochastic neighbor embedding. The effectiveness and stability of the proposed model are validated through the experiments. With the proposed method, different fault types can be precisely identified and high classification accuracy is achieved in a hydraulic axial piston pump.

## 1. Introduction

Hydraulic transmission systems are broadly used in state-of-the-art machinery because of their strengths in terms of high energy, quick response, easy control, high output force [1,2,3]. As the pivotal energy conversion component of hydraulic transmission system, a hydraulic axial piston pump plays a crucial role in guaranteeing the stability of the system in many fields. Owing to the unpleasant operational environment and highly intensive conditions, the inevitable failure may result in the breakdown of the whole system and even massive losses [4,5,6]. In the light of the concealment, complexity and the disparate causes of faults, it is challenging and of great significance to implement precise and efficacious fault diagnosis of hydraulic axial piston pump to enhance the stability of the system.

Immense amounts of research on machinery fault diagnosis have concentrated on the common diagnostic methods [7,8,9]. Conventional approaches pay more close attention to the mechanism analyzing and the acquisition of the characteristic frequency. Owing to the limited characteristics as well as the complex structure of pump itself, it is hard to accurately diagnose different types of faults only from the experts’ subjective experience and existing knowledge. As a typical representative in the progress of artificial intelligence, deep leaning-based technologies have drawn tremendous attention in the fields of intelligent fault diagnosis [10,11,12]. In place of the great dependence of traditional methods on the previous knowledge and experience, intelligent approaches accomplish the automatic feature extraction from the input signals. Li et al. proposed a support vector machine (SVM) framework integrating an improved multiscale permutation entropy towards the fault diagnosis of a bearing [13]. Combining a multiscale permutation entropy algorithm and Mahalanobis distance, a new SVM was developed for fault diagnosis of a wind turbine rolling bearings. It was worth pointing out that the beetle antennae search was used in the classification stage to promote the classification of the SVM [14]. On account of the limited and imbalanced fault data and the shortcomings of the conventional SVM, Wei et al. employed an oversampling strategy for bearing fault diagnosis. Meanwhile, an optimization algorithm called moth-flame was used in the final classification [15]. The investigations above conducted fault diagnosis with the shallow network models, in which it is hard to accomplish the precise classification of the more complicated conditions [16,17].

Deep learning (DL)-based methods effectively overcome the disadvantages of the common learning model in the feature extraction and can automatically extract the useful information from raw input data [18,19,20]. Owing to the capability of automatic learning, DL-based technologies have been successfully used for machinery fault diagnosis. Motivated by the domain adaption, a new multi-mask deep learning model was established for gearbox fault diagnosis and achieved the challenging transfer of a trained DL model to industrial applications [21]. Zhong et al. constructed a convolutional neural network (CNN) model on the basis of transfer learning (TL) for fault diagnosis of a gas turbine [22]. Li et al. exploited a TL-based model for rolling bearing fault diagnosis [23]. In consideration of the insufficient fault data and the diversified working conditions, a novel intelligent method was developed based on TL and a multiwavelet auto-encoder [24]. Guo et al. employed a convolutional neural network (CNN) for rotor fault diagnosis and conducted continuous wavelet transform (CWT) for time-frequency transformation [25]. The proposed deep model was demonstrated to be universal and can be extended to other conditions of fault diagnosis. Similarly, Xu et al. combined CWT and CNN for rolling bearing fault diagnosis and gained desirable classification performance [26]. Liang et al. developed a compound network model for fault diagnosis of a rolling bearing and gearbox, integrating CNN, generative adversarial net and WT [27]. Analytical wavelet transform was utilized for signal-to-image transformation, and an enhanced CNN was employed for detect identification of centrifugal pump [28]. It is noteworthy that many researchers apply acoustic signal in place of the commonly-used vibration signal. By using Hilbert–Huang transform processing of the acquired raw signal, Gao et al. carried out a CNN model for sensor fault diagnosis [29]. In order to accomplish cross-domain fault diagnosis, a deep domain adaptation model named the double-level adversarial domain adaptation network was built for a bearing and planetary gearbox [30]. Specifically, feature extraction was completed on the domain-level, and the classification was performed on the class-level simultaneously. Based on an adversarial idea, a deep semi-supervised learning model was employed for fault diagnosis of the transmission and bearing [31]. Jia et al. employed a CNN with normalization to overcome the data imbalance and interpreted the learning process by visualization [32]. Instead of the information extraction from the single channel, multi-channel signals were taken as the input of transfer CNN for bearing and gear fault diagnosis [33]. The researches on the deep model-based intelligent fault diagnosis have been concentrated on the applications in the bearing, gearing and gearbox. However, few investigations on pump are conducted, and the studies are seldom performed on hydraulic axial piston pump. In light of complicated structures, changeable operation conditions and challenging data acquisition, the accurate and effective fault diagnosis is immensely difficult for a hydraulic axial piston pump.

In this study, three important contributions are made as follows:

(1) The proposed diagnosis method can compensate for the deficiencies of the conventional fault diagnosis methods, and will provide a solid foundation for the exploitation of the novel intelligent methods for a hydraulic pump.
(2) The structure of the proposed model is greatly simplified. The deep model in this research only includes two convolutional layers, and the corresponding parameters to be trained are greatly reduced. In comparison to the traditional LeNet 5, the number of the channels and the input size of the images are improved to enhance the performance of the diagnostic method.
(3) In light of the structural simplicity and fast computation of the method, it is more conducive to the practical operation and application.

This work is based on the deep model-based fault diagnosis approach of research hydraulic axial piston pump. In Section 2, the basic principles of CNN and CWT are briefly illustrated. The data preprocessing and the structure of the CNN are detailed in Section 3. In Section 4, the effectiveness of the proposed CNN model is demonstrated through the experimental data of hydraulic axial piston pump. Finally, the conclusions are presented and prospects for future work are outlined in Section 5.

## 2. Basic Theory

### 2.1. Brief Introduction of Convolutional Neural Network (CNN)

In the face of the present dramatic machinery fault data, the intelligent methods based on deep learning present superior condition monitoring and fault diagnosis [34,35,36]. Among the numerous deep learning models, CNN is considered to be one of the most popular and effective networks. It is capable of automatically learning useful feature information from the non-linear and non-stationary signals [37,38]. There are three primary strengths for CNN models, local connection, weights sharing and down-sampling, respectively, which can be distinguished from other deep models. Therefore, CNN has aroused great attention in many types of task, such as 1D time series and 2D image data. In particular, CNN is powerful in image processing and pattern recognition.

Compared with traditional fully connected neural networks, the relatively fewer parameters make it easier to train and optimize CNN. Similar to the annual neural network, the input and output layers are involved in CNN. The input layer is utilized to store the transformed array from time series or images. In addition to this, typical CNN structure still includes a convolution layer, a rectified linear unit (ReLU) layer called the activation layer, and a pooling layer. The feature extraction is accomplished by the convolution layers from raw input. The selection of the effective features is performed by pooling layers. Hence, the special structure of CNN desirably achieves the integration of feature extraction and classification, which is the advantage in comparison to the conventional fault-diagnosis methods.

#### 2.1.1. Convolution Layer

There are many convolutional kernels called filters in convolution layer, which is taken as the feature extractor. The convolution is carried out by the convolutional kernel and the input array to gain the feature matrix of the next layer. The number of convolutional kernels in the next layer is equal to the amount of output of the front convolutional layer. The size of the convolutional kernels is the same as the local receptive field in the convolutional layer [39,40].

The output feature map of the convolutional layer can be represented by:

(1) $$x_{j}^{l}=f(\sum_{i\in M_{j}}x_{i}^{l-1}\times k_{j}^{l}+b_{j}^{l})$$

where, (×) represents the convolution operation. The input of the model is denoted by x. $k_{j}^{}$ is convolutional kernel. The corresponding bias of $b_{}^{}$ is introduced in the convolution process. In order to enhance the nonlinearity of the CNN model, activation function f is used. 

#### 2.1.2. Rectified Linear Unit (ReLU) Layer

ReLU is a widely-used activation function in annual neural network. It is generally considered as a non-linear function. It is performed on the output of the convolution layer. The establishment of the ReLU layer leads to the sparsity of the network. The interdependence of parameters can be reduced, which effectively alleviates the occurrence of overfitting in the model [41].

#### 2.1.3. Pooling Layer

The pooling layer, namely down-sampling layer, is usually assigned after the convolutional layer to reduce the size of the input feature map and decrease the number of parameters in the network model [42,43]. Meanwhile, shift-invariant is also maintained. Therefore, it is beneficial to govern the overfitting of the model and decrease the training time. The generalization ability of the CNN model can be promoted by pooling operation. It is worth mentioning that no parameters are requested to be learned in the pooling layer.

The process of pooling operation can be expressed by,

(2) $$a_{j-s}^{l}=f(w_{j}^{l}down(M_{j}^{l-1})+b_{j}^{l})$$

Among these, down(⋅) denotes the pooling operation on the obtained features from the previous convolutional layer, usually the computation of the maximum or the mean values. In the function f, $M_{j}$, $w_{j}^{l}$, and $b_{j}^{l}$ denote the feature map, the weight and the bias.

Generally, there are three methods of subsampling, including average-pooling, max-pooling and stochastic-pooling. The error of feature extraction is mainly attributed to the variance of estimated value from the limited neighborhood size and parameter error of the convolutional layer. Average-pooling can reduce the first kind of error, and retain more background information of the image. Max-pooling can reduce the second kind of error, and retain more information on the texture of image. Stochastic-pooling criteria are between these two, and assign probabilities to pixel points according to their numerical size; and then subsampling is performed according to the probability. Specifically, the criteria are approximate to the average-pooling in the average sense and comply with the max-pooling in the local sense.

#### 2.1.4. Fully Connected Layer

In the fully connected layer, the dimensionality of layers changes compared with the previous layers. Like common annual neural networks, the activations from all the above layers are connected. In consideration of the simplicity and effectiveness of the calculation, the softmax regression function is usually employed to achieve the final fault classification from the useful features obtained. 

### 2.2. Continuous Wavelet Transform

On account of the limitations of one-dimensional (1D) time-domain/frequency-domain analysis, two-dimensional (2D) processing methods present advantages in analyzing non-linear signals. Many time-frequency analysis methods have been employed to process time series signals, involving short-time Fourier transform (STFT), Hilbert–Huang transform (HHT) and continuous wavelet transform (CWT) [44,45,46]. Localization can be accomplished via STFT and WT in both time and frequency domains. Compared with STFT, the superiority of CWT is that it carries out analysis in a variable time-frequency window instead of only the fixed window [47,48]. 

On the basis of the advantages of CWT in signal processing, CWT is used for time-frequency transformation in this work. The fundamental theory of WT can refer to the relative investigations [49,50,51]. The family of the mother wavelet is considered as the translated and scaled single mother wavelet, and it can be expressed in the following,

(3) $$\psi_{u,s}(t)=\frac{1}{\sqrt{s}}\psi (\frac{t-u}{s})$$

Among these, $\left\{\psi_{u,s}\right\}$ denotes the mother wavelet generated by a single wavelet; s and u are two different variables; s denotes the scale; u presents the shift along time and u∈R, which is used to govern the translation of the wavelet function. In order to make $\psi_{u,s}(t)$. independent of s and u, normalization is carried out by $\frac{1}{\sqrt{s}}$.

Correspondingly, the WT can be presented as follows,

(4) $$W_{f}(u,s)=\langle f,\psi_{u,s}\rangle =\frac{1}{\sqrt{s}}\int x\left(t\right)\psi (\frac{t-u}{s})dt$$

Wavelet analysis is employed to measure the similarity between the basis functions (wavelets) and the original function. The wavelet transform coefficients reflect the relevancy of the function and the daughter wavelet at the selected scale.

## 3. Proposed Intelligent Fault-Diagnosis Method

### 3.1. Data Description

**Figure 1:**
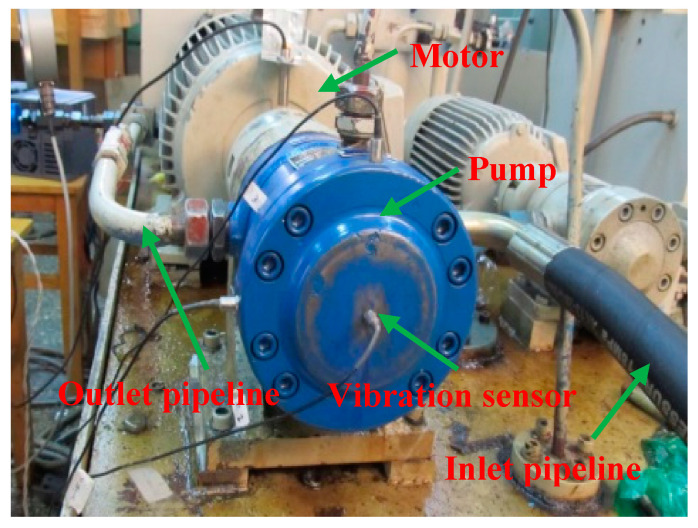
The test platform for fault diagnosis.

The experiments were carried out on the hydraulic axial piston pump test bed, as shown in Figure 1. The test bench mainly includes a motor, a pump and a vibration sensor. The primary structure in this testbed is a swash-plate axial piston pump, involving seven plungers. The rated speed of the axial piston pump is set as 1470 r/min. Equivalently, the rotary frequency is 24.5 Hz. The experimental data collection is carried out in Yanshan University. The frequency of sampling is taken as 10 kHz. 

Vibration signals are obtained through simulating five various conditions, including zc, xp, sx, hx and th. The gained vibration data were employed for data preprocessing and the following intelligent fault diagnosis. The detailed descriptions of five health conditions and the corresponding category labels are depicted in Table 1.

### 3.2. Data Preprocessing

As important technologies in machinery fault diagnosis, data preprocessing methods are generally used to extract feature information from raw signals [52,53]. Intelligent diagnosis methods integrate signal acquisition, feature extraction and final classification, and present remarkable advantage in accomplishing machinery fault classification [54,55]. The methods based on deep network models request special data input with regard to the 2D input and the amount of training datasets [56,57].

**Figure 2:**
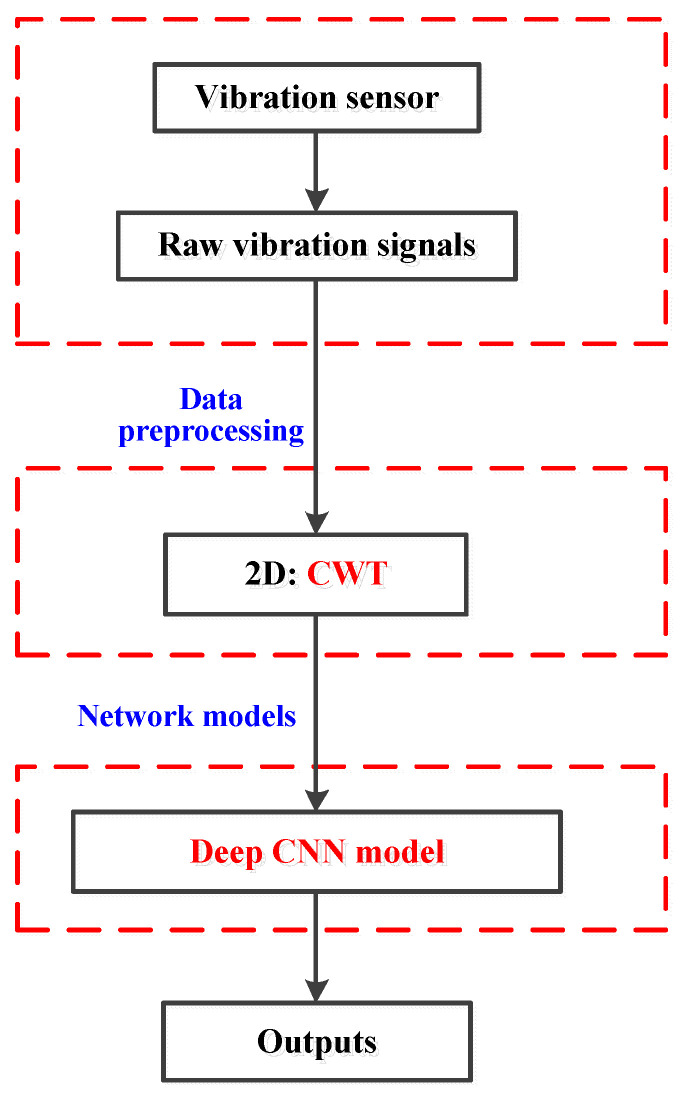
Flowchart of data preprocessing for vibration signals.

In this research, a 2D image input is required for 2D CNN. The specific flowchart is displayed in Figure 2. In the first, raw vibration signals of each fault type are split into different data segments. Each segment involves 1024 sampling points. Then, according to the input requirements of deep models, various data preprocessing methods can be used to achieve the transformation of the images. Many diverse preprocessing methods have been employed to convert raw signals into time-frequency images, including CWT, S-transform, cyclic spectral coherence and so on [58,59,60]. As a typical signal-processing method, CWT is selected to the time-frequency analysis for the construction of useful fault information. A raw vibration signal can be transformed into a 2D wavelet coefficient matrix via CWT. ComplexMorlet has been demonstrated to be effective for fault diagnosis and can be matched with the response of the fault signals [61]. The obtained images are fed into the CNN model. The parameters of the CNN model are established through the training process using training datasets. It is worth noting that the training loss and the testing accuracy are exploited for evaluating the performance of the model.

### 3.3. Proposed CNN-Based Intelligent Diagnosis Method

In consideration of the spatial correlation of images, a CNN model called LeNet 5 was constructed for image processing [62]. On the basis of LeNet 5, an improved CNN model is employed for intelligent fault diagnosis of the hydraulic pump. The steps of the method are as follows: the vibration signal is acquired by using the sensors. Then the acquired signals are converted into images and split into training datasets and testing datasets. Furthermore, the CNN model is established and trained with the training datasets. Finally, the classification performance of the model is verified by using the testing datasets as input.

**Figure 3:**
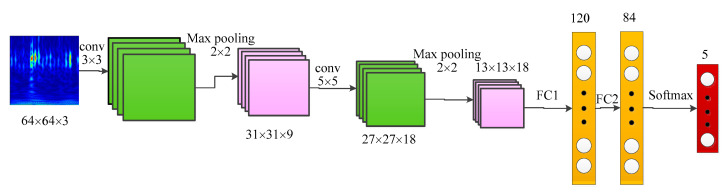
The structure of the proposed convolutional neural network (CNN) model.

The structure of the deep model is constructed with two alternative convolutional layers (Conv) and sub-sampling layers. Then three fully connected layers (FC) are included for the accomplishment of the classification (Figure 3).

In order to decrease the parameters and the dimension of features, maxpooling is employed in the model. With regard to each maxpooling layer, the size of pooling area is taken as 2 × 2. As there are five different fault types for hydraulic axial piston pump to classify, the output of the CNN model is taken as 5. Finally, the softmax function is chosen for the classification stage.

## 4. Verification of Proposed CNN Model

### 4.1. Input Data Description

For the vibration signals of each health condition, the image conversion was performed using CWT. On account of the remarkable advantages of ComplexMorlet in comparison with regular wavelets, it was chosen as wavelet basis function during the time-frequency processing. The parameters of CWT were the following: the bandwidth and the center frequency were set as 3, and the length of the scale sequence was taken as 256.

**Figure 4:**
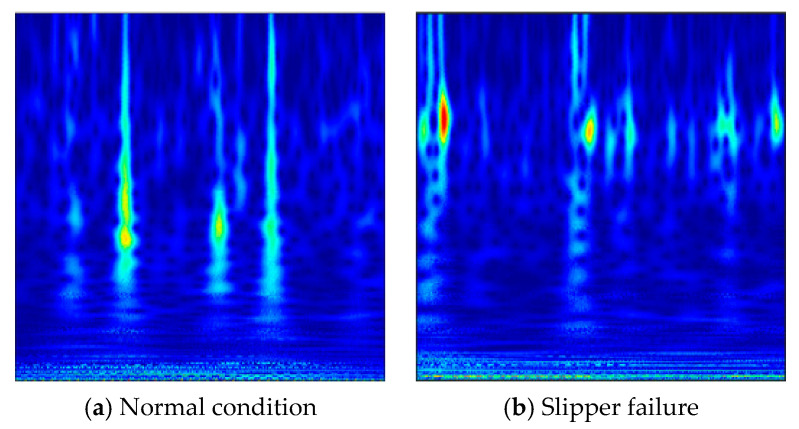

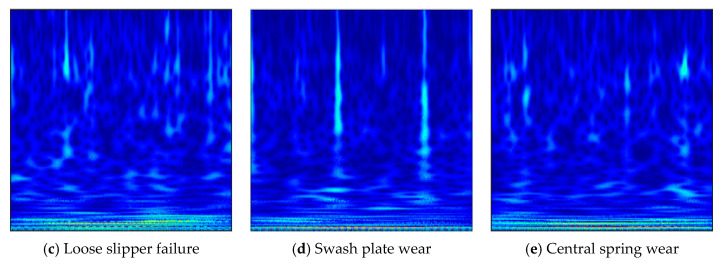
Time-frequency representations under 5 health conditions.

The time-frequency distributions of five health conditions are depicted in Figure 4. It can be found that there were no marked differences for the time-frequency representations of different fault categories. Although the frequencies of different conditions changed with time, it is still tough to achieve the identification of diverse fault types only on the basis of the current observed apparent characteristics. This reflects the limitations of traditional diagnostic approaches mainly dependent on the experience and knowledge in another aspect. Furthermore, it provides a great prospect for the deep CNN model in automatic learning of useful fault features from such similar visualized images and precise classification.

As for the transformed time-frequency distribution images, a total of 6000 samples are gained for the establishment of the datasets. There are 1200 images in each category of fault. Owing to the difference between the obtained raw image and the input of the CNN model, the operation of data transform was performed in the image processing. The image size was transformed into the same size of 64 × 64. It should be pointed out that the samples in the training dataset were processed with the random horizontal flip, while the testing samples were not. In consideration of the possible influence of the input data on the training of the model, the ratio of 7:3 was selected in randomly splitting the training dataset and testing dataset. For each fault category, 840 training samples and 360 testing samples were acquired respectively. In the training of the model, the type labels were set for each condition. The descriptions of data were displayed in Table 2. Moreover, only the training samples were employed for updating and confirming the parameters of the model in the training process. The test samples were exposed to the model until the testing stage began.

### 4.2. Parameter Selection for the Proposed Model

There are two types of parameter in machine-learning models, the parameters obtained via the learning and evaluation of training data and tuned parameters through artificial setting, respectively. The latter is generally called hyperparameter, which is considered as the parameter of the parameter. Changing it will lead to training the model again. Since the hyperparameters have a great effect on the classification performance of the model, its different settings are significant for the building of the model. The following hyperparameters were analyzed and discussed, involving epoch, batch size, the number of convolutional kernel and the size of convolutional kernel. A suitable network model will be established via the optimization of the parameters above.

The epoch can be referred to the number of times that all the training samples are input into the neural network for training. The selection of a proper epoch will be conducive to the fitting and classification of the model. Too small an epoch may be harmful to the fitting of the network model. Likewise, too big an epoch will result in the increase of computing cost although it can promote classification accuracy.

**Figure 5:**
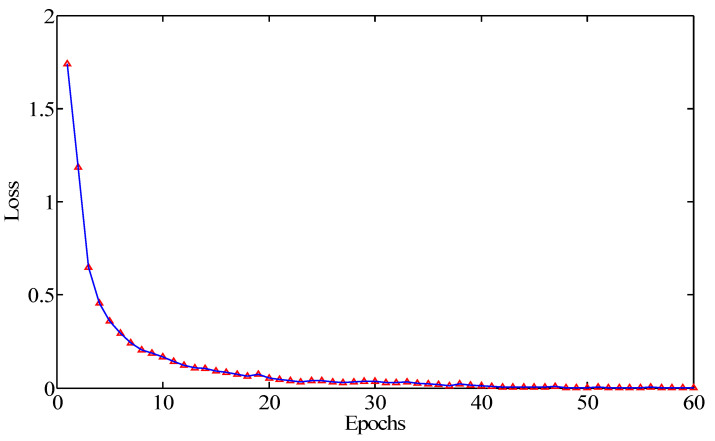
The training loss of the proposed model.

In this work, we take the epoch as 60 and repeated the trials 10 times to investigate the convergence condition of the network model. The average values of 10 trials were recorded as the final training loss and testing accuracy. Seen from Figure 5, the training loss was above 1.5 at the beginning of the training. When the epoch was less than 10, it rapidly decreased with the increase of the epochs. Sequentially, the loss slowly decreased. When the epoch was more than 40, it approached a very small value and maintained steady.

**Figure 6:**
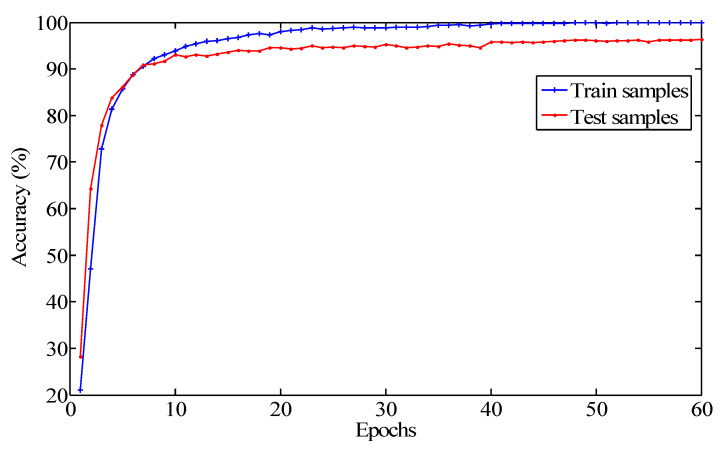
The curve of the classification accuracy of the proposed model.

As shown in Figure 6, contrary to the change tendency of the training loss, there was a trend of increase for the classification accuracy with the increase of training epochs. Before the epoch increased to 10, it soared to around 90%. Subsequently, there was a subtle increase with the further increase of the epochs. When the epoch was more than 20, the accuracy of the testing dataset reached above 94%. When the epoch was up to 40, the testing accuracy exceeded 96%. In addition, the accuracy presented a gentle fluctuation in the following training process. It can be implied that the network model achieved convergence after 40 epochs. 

Batch size can be understood as the sample size selected randomly each time during the training process of the model. When samples of a batch size are trained, the weight and bias of the network are accordingly updated once. Similar to the epochs, too small or large a batch size may not be beneficial for the classification performance of the model. Hence, it is of great importance to select the suitable batch size in the training of the network model.

Through considering the factors of the sensitivity of the GPU to the batch size value of 2^n^, 8 multiples, the selection of batch size was explored. Finally, the batch size was taken as 42.

The number of convolutional kernel means the number of the extracted features. If the number of convolution kernels is insufficient, the feature extraction is insufficient. It is difficult to obtain the ideal classification accuracy. If there are too many convolution kernels, too many parameters of the neural network need to be trained and will result in the increase of computational cost. The number of the convolutional kernel in this study is selected by setting the number of the second convolutional layers as the multiples of the first layer.

The larger convolution kernel size represents the more weights in the convolution kernel, and the automatic learning ability of the network is stronger. However, the increase of weights leads to the increase of training parameters and incurs computational costs. The selection of the convolution kernel size in this research is based on the small and deep principle in deep learning.

**Figure 7:**
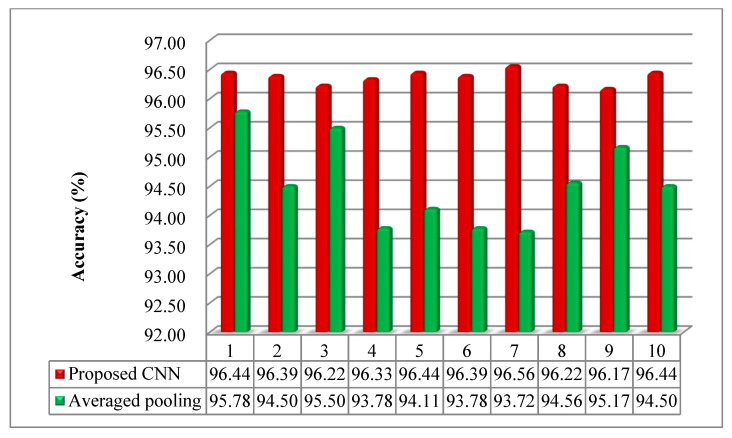
The testing accuracy with maxpooling and average pooling.

The pooling layer can reduce the amounts of the parameters and calculated quantity, and then effectively govern the overfitting of the model. The operation of the average pooling was used to compare with the maxpooling in the proposed model. It can be seen from Figure 7 that the accuracy of the model with maxpooling strategy was more than that with average pooling. The accuracy of the model was over 96%, and the results of 10 trials presented a slight change. On the contrary, the accuracy was mostly less than 95%, moreover, the difference of each trial was more obvious. It can be indicated that the maxpooling was more conducive to the model in dimensionality reduction.

### 4.3. Performance Validation of the Proposed Model

In order to verify the robustness and availability of the proposed CNN model, 10 repeated trials were carried out via the parameters above. Maximum pooling was used to reduce the dimension of the learned features. SGD optimizer was used for the optimization of the model. The original learning rate was set to be 0.009, and the momentum was set as 0.9.

**Figure 8:**
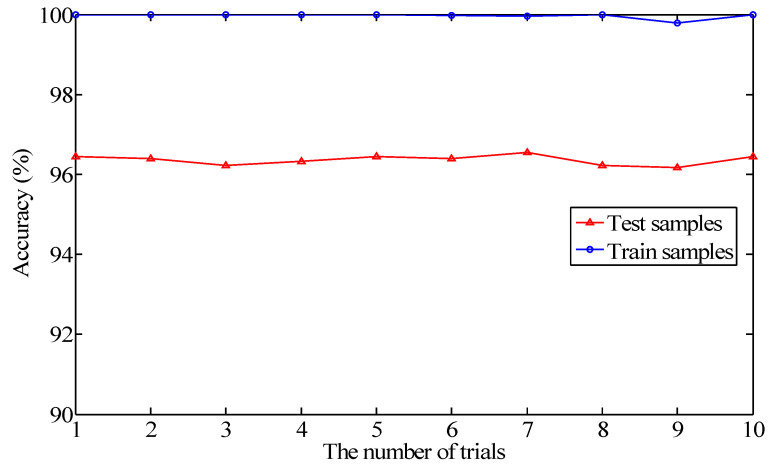
The accuracy curve of 10 trials on the training and testing samples.

As can be observed in Figure 8, the training accuracy was up to around 100%, indicating that the model reached convergence. Compared with the testing and training accuracy, there is a certain difference. However, the testing accuracy of 10 trials was more stable and all surpassed 96%. Therefore, it can be demonstrated that the proposed model possessed the effectiveness for fault diagnosis of a hydraulic pump.

**Figure 9:**
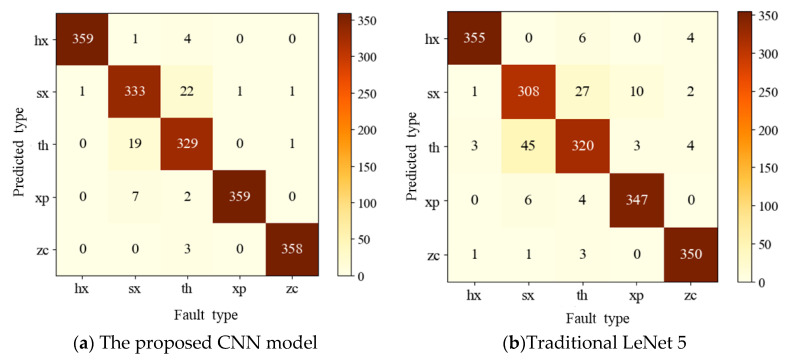
The confusion matrix of the testing samples on CNN in the seventh trial.

In order to simply and intuitively evaluate the classification accuracy of the model, a confusion matrix is generally used as an effective statistic tool as well as a visualized method in data mining and analysis [63,64,65]. Hence, a confusion matrix was carried out to analyze and evaluate the classification results towards each fault type. On account of the non-stationary vibration signals of a hydraulic pump, the CNN constructed proved to be effective and precise for the classification of different types of fault. As can be seen from Figure 9a, the sample number correctly classified was around 360 to predict the type of hx, xp and zc. In regard to another two conditions of th and sx, there were a different number of misclassifications. Nineteen samples in the sx were misclassified into the condition of th. As for the condition of th, 22 samples were misclassified as sx. It can be revealed that the model presents a certain deficiency in the classification of the fault types of th and sx. It could be explained that the information from the time-frequency images of th and sx provides the features with some similarity to the CNN model. The two types may be easily confused, and it makes it difficult for the model to differentiate one from the other according to the present extracted feature information. Compared with the proposed model, a more severe misclassification phenomenon was observed in the results of traditional LeNet 5. As shown in Figure 9b, especially in the conditions of th and sx, there were as many as 45 samples misclassified into the type of th. Meanwhile, 27 samples in the type of th were confused for sx. By contrast with the proposed model, the classification of the xp was not very satisfactory.

**Figure 10:**
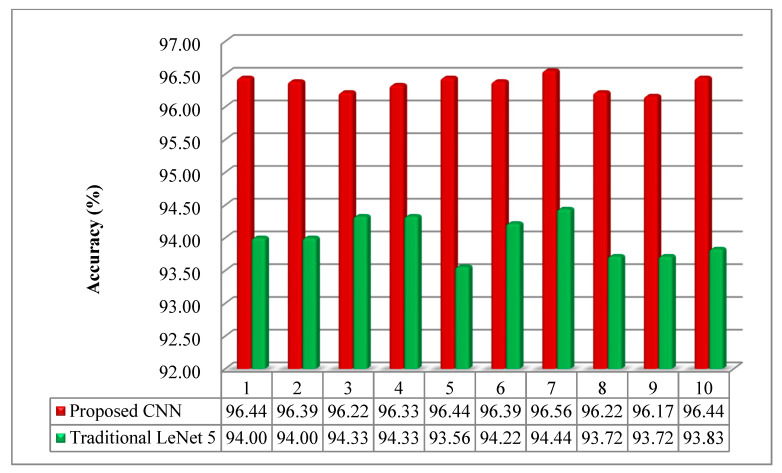
The comparison between the proposed CNN and traditional LeNet 5.

Compared with the traditional LeNet 5 CNN model, the proposed CNN model displayed the enhanced performance and advantageous stability. As depicted in Figure 10, the classification only reached around 94% through adopting LeNet 5. The proposed CNN achieved more than 96% and the results of 10 trials were stable.

Neural network models are generally considered a black box. This seems to be very mysterious and unsearchable within the network. The successes of deep neural networks in many fields indicate that they may be learning hidden representations automatically. Hence, it is meaningful to exploit the internal operations of CNN model and make it possible to transform the intricate information into the interpretable features.

The visualization of the learned features can shed light on the potential characteristics of the objects to be classified. In order to achieve the non-linear dimension reduction, *t*-distributed stochastic neighbor embedding (*t*-SNE) is regarded as an effective algorithm to decipher the high-dimensional feature representations [66]. Through affinity transformations, data points can be mapped to probability distributions. The feature extraction of convolutional layers and fully connected layers were employed for the research on the learning efficacy of the CNN model, Conv 1, Conv 2, FC 1, FC 2, and FC 3, respectively. In addition, the reduction result of the original input was taken into account.

The first two dimensions of the reduced features were selected for the analysis and discussion. Specifically, the high-dimensional feature representations were reduced to two dimensions. A testing sample was represented by each reduced point via *t*-SNE. The dimensions of *t*-SNE are shown with the horizontal and vertical axes, respectively. 

**Figure 11:**
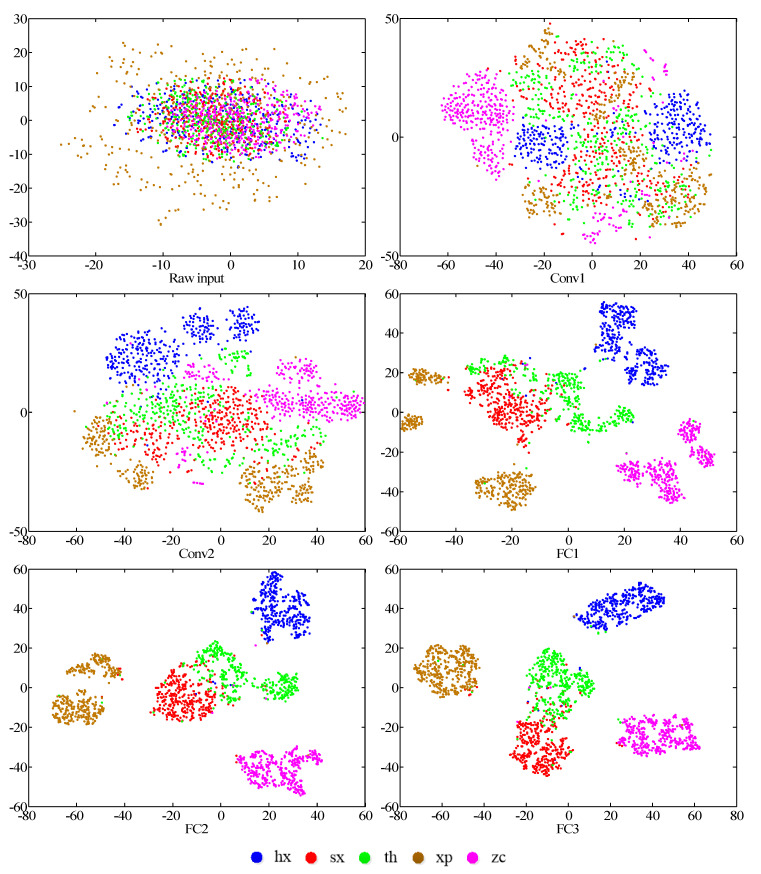
Visualization of different layers via *t*-distributed stochastic neighbor embedding (*t*-SNE): feature representations for the raw input, five convolutional layers and the last fully connected layer respectively.

From Figure 11, it can be observed that the features of different fault types become much easier to distinguish with the increase of the network layers. It can be indicated that the potential features are acquired through the learning of the model.

As for the representations of the original input, a uniform distribution emerges for five types of fault. It is not practical to distinguish each fault type in this step. Through convolutional operation of Conv 1 in the model, the features of type zc and hx begin to come together, while dispersed distribution is found in the other three categories. As shown in Conv 2, the features of type xp and sx gradually cluster into one area, but the overlap of various features is still apparent. Through the two layers of convolutional operation and FC layers, the features of all the types are not the initial scattered points, and the same types cluster into a special region. The classification of different fault types is becoming clear and divisive. However, the overlay of the type sx and th can be found. The misclassification of the two types implies that the model is not sensitive in identifying the time-frequency images of both types of fault. In the meantime, the visualization results are inconsistent with those obtained from the metrics of the confusion matrix. With the feature learning of the model, the abstract representations are gained and can provide effective information for the classification of faults.

## 5. Conclusions

CWT is advantageous for non-stationary signal preprocessing. CNN is predominant in image identification and classification. Combining the strengths of CWT and CNN, a new fault diagnosis approach is exploited to accomplish the extraction of useful feature and fault classification toward a hydraulic axial piston pump.

The experimental results show that the proposed CNN model can accurately classify the types of fault in the states of zc, hx and xp. The corresponding classification accuracy reaches more that 99%. Although there is a misclassification in another two conditions of hx and th, the testing average accuracy is above 96%. The potential reason could be that it is hard for the CNN model with regard to the indistinguishable parts in the two time-frequency images. The results of the repetitive trials confirm the stability of the proposed deep model. The reduction of features is carried out by *t*-SNE, including the effect of different network layers. The visualization results show that five different types of fault are efficaciously identified through the learning of the model. Hence, the proposed intelligent method can adaptively acquire the fault features from the input images. It can realize automatic learning and compensate for the deficiency of conventional fault-diagnosis methods.

In this research, the time-frequency distributions may be hard to distinguish for the CNN model in view of the types of hx and th. In future, different data preprocessing methods will be employed for the enhancement of the initial feature extraction. Presently, the vibration signal is selected for the feature extraction of faults, and there is similarity in the converted images of the two types of fault. In addition, the acoustic signal will be investigated to explore the diagnostic performance of the deep model towards a hydraulic axial piston pump.

## Figures and Tables

**Table 1:** The operation conditions and category labels of a hydraulic axial piston pump.

Health Condition	Description	Index	Type Label
Normal	no any fault in hydraulic pump	zc	0
Faulty	swash plate wear	xp	1
	loose slipper failure	sx	2
	slipper wear	hx	3
	central spring wear	th	4

**Table 2:** The number and labels configuration of datasets for hydraulic axial piston pump under 5 conditions.

Fault Type	Train Dataset	Test Dataset	Type Label
hx	840	360	0
sx	840	360	1
th	840	360	2
xp	840	360	3
zc	840	360	4
total	4200	1800	—
